# Membrane insertion of the BAX core, but not latch domain, drives apoptotic pore formation

**DOI:** 10.1038/s41598-017-16384-4

**Published:** 2017-11-24

**Authors:** Hector Flores-Romero, Miguel Garcia-Porras, Gorka Basañez

**Affiliations:** Biofisika Institute (CSIC, UPV/EHU), Barrio Sarriena s/n, Leioa, 48940 Spain

## Abstract

Despite intensive research effort, how the paradigmatic proapoptotic protein BAX forms lethal apoptotic pores at the mitochondrial outer membrane (MOM) remains incompletely understood. Here, we used biophysical tools and minimalist model systems to identify the specific regions in BAX driving apoptotic pore formation, and to gain more insight into underlying mechanisms. Fluorescence mapping revealed that fully active BAX adopts a BH3-in-groove dimeric conformation in MOM-like membranes, with BAX α4-α5 helices belonging to its core domain inserting deeper into the membrane lipid bilayer than BAX α6-α8 helices belonging to its latch domain. In our reconstituted systems, antiapoptotic BCLXL formed canonical heterodimeric BH3-in-groove complexes with BAX, and blocked membrane insertion of BAX core α4-α5 helices, but not BAX latch α6-α8 helices. Moreover, poly(ethylene glycol) (PEG) conjugation (PEGylation) at multiple individual sites along the BAX core, but not latch domain, potently inhibited BAX pore-forming activity. Additional combined computational and experimental evidence revealed that the BAX core α5 helix displays a bilayer-destabilizing membrane interaction mode that is absent in BAX latch α6-α8 helices. Based on this collective set of evidence, we propose that membrane insertion of the BAX core, but not latch domain, is critical for BAX apoptotic pore formation.

## Introduction

BAX is a key proapoptotic member of the BCL2 family which kills cells by forming an atypical pore at the mitochondrial outer membrane (MOM)^1^. This apoptotic pore is essential for allowing the release of cytochrome c (cyt c) and other mitochondrial apoptogenic factors into the cytosol, and is considered the pivotal step in the mitochondrial apoptotic pathway^2^. It is well established that BAX exists in an inactive conformation in healthy cells, and that functional BAX activation is tightly regulated by two other factions of the BCL2 family. On the one hand, proapoptotic BH3-only proteins such as cBID trigger activation of BAX at the MOM through a complex set of conformational changes culminating with formation of the BAX apoptotic pore. On the other hand, antiapoptotic BCL2-type proteins, including BCL2 itself and BCLXL, principally act by sequestering proapoptotic BCL2 family partners in stable protein complexes at the MOM, thereby blocking apoptotic pore formation.

According to current knowledge, apoptotic pore formation relies in two distinct but interlinked biochemical activities of BAX: (1) homo-oligomerization, which is mainly mediated through BAX:BAX protein-protein interactions, and (2) perturbation of membrane structure, which primarily, if not solely, occurs through BAX:lipid interactions. Structural and functional studies have provided important insight into this dual activity of BAX. Early NMR studies revealed that inactive BAX forms a tight globular helical bundle fold comprising nine α-helices^3^. One critical feature of the inactive BAX structure is the presence of an extended hydrophobic groove mainly comprising the BAX α3-α5 region that is also present in its close homologue BAK, as well as in all antiapoptotic BCL2-type molecules^2^. Such canonical BCL2 hydrophobic grooves share the capacity to act as “receptors” for binding proapoptotic “BH3-ligand” domains, although other non-canonical protein:protein interaction modes between BCL2 family proteins have also been identified^4^. Importantly, both the canonical BAX “receptor” groove and the BAX “BH3-ligand” domain (BAX α2) remain masked in the inactive BAX conformation, and together constitute the so-called BAX core domain^2^. Another salient characteristic of the inactive BAX conformation is the presence of hidden hydrophobic residues with potential for membrane interaction and/or perturbation that are localized in multiple helices of the protein.

It is becoming clear that following binding by BH3-only activator ligands such as cBID, the BAX core domain (α2-α5) dissociates from the so-called BAX latch domain (α6-α8), as well as from the BAX N-terminal (α1) and C-terminal (α9) helices^5,6^. Nevertheless, the specific regions in the BAX molecule that drive apoptotic pore formation via BAX:BAX and BAX:lipid interactions remain ill defined. The X-ray crystal structure of a truncated GFP-BAX fusion construct comprising the entire BAX core domain provided strength to the view that the assembly of a BH3-in-groove BAX dimer constitutes a pivotal step in the molecular pathway for BAX activation^5^. However, it remains unclear whether this crystallographic BAX core dimer structure faithfully represents the conformation adopted by active BAX in its native membrane environment. In fact, under certain apoptotic conditions, alternative BAX dimeric conformations have been described at the MOM level^7,8^. Additionally, how dimeric BAX species grow into higher order oligomers is not well understood, since multiple different interdimer interfaces have been identified in BAX and its close homologue BAK^7–13^. Indeed, even the molecularity of BAX/BAK required to form functional apoptotic pores remains undetermined^14–18^.

From the perspective of BAX:lipid interactions implicated in apoptotic pore formation, initial studies attributed a critical role to insertion of the BAX α5-α6 region into the MOM lipid bilayer as a transmembrane (TM) helical hairpin, akin to proteinaceous channel-like models proposed to explain the action of colicins^19^. Recent work, however, challenged this view by providing evidence that upon functional BAX activation, the BAX α5 and α6 helices: (i) dissociate from each other, rather than maintaining a hairpin configuration^5^; and (ii) adopt a surface-parallel, rather than TM orientation^20^. Based in these observations a new model emerged where the concerted shallow insertion of BAX α5 and α6 helices into the MOM elicits the formation of a proteolipidic apoptotic pore through destabilization of the MOM lipid bilayer structure. It has also been proposed that additional helices of the BAX core (α4)^5^, latch (α7, α8)^11,20^, and C-terminal domains (α9)^8^ actively drive BAX proteolipidic pore formation through shallow membrane insertion and bilayer destabilization. However, despite the proteolipidic nature of the BAX apoptotic pore has been debated for more than a decade^14–25^, the exact membrane topology of individual BAX helices, and the extent to which membrane immersion of defined BAX regions contributes to BAX pore formation remain incompletely delineated. On top of this, the specific protein:protein and protein:lipid interaction mechanisms through which antiapoptotic proteins such as BCLXL block BAX apoptotic pore formation are still under investigation^26–33^.

Here, we used physiologically-relevant model systems and biophysical and biochemical tools to analyze the membrane topology of individual helices of BAX core and latch domains, as well as their specific contribution to BAX pore-forming activity. Fluorescence mapping studies showed that cBID-activated BAX adopts a BH3-in-groove dimeric conformation in MOM-like membranes, with BAX core α4-α5 helices inserting deeper into the membrane hydrophobic core than BAX latch α6-α8 helices. In our reconstituted systems, antiapoptotic BCLXL inhibited both membrane insertion of BAX core α4-α5 helices and BAX pore-forming activity via canonical BH3-in-groove heterodimeric interactions. We also showed that PEGylation of multiple sites along the BAX core, but not latch domain, inhibits BAX membrane-permeabilizing activity. Moreover, combined computational and experimental evidence indicated that the isolated BAX core α5 helix displays a mode of interaction with the membrane that destabilizes its lipid bilayer structure, which is unlike the case of the isolated BAX latch α6 and α7-α8 helices. Based on this collective set of evidence, we propose that insertion of the core, but not latch domain, of BAX into the MOM lipid bilayer actively contributes to BAX apoptotic pore formation.

## Results

### Functional and structural analysis of recombinant BAX monocysteine mutants

**Figure 1:**
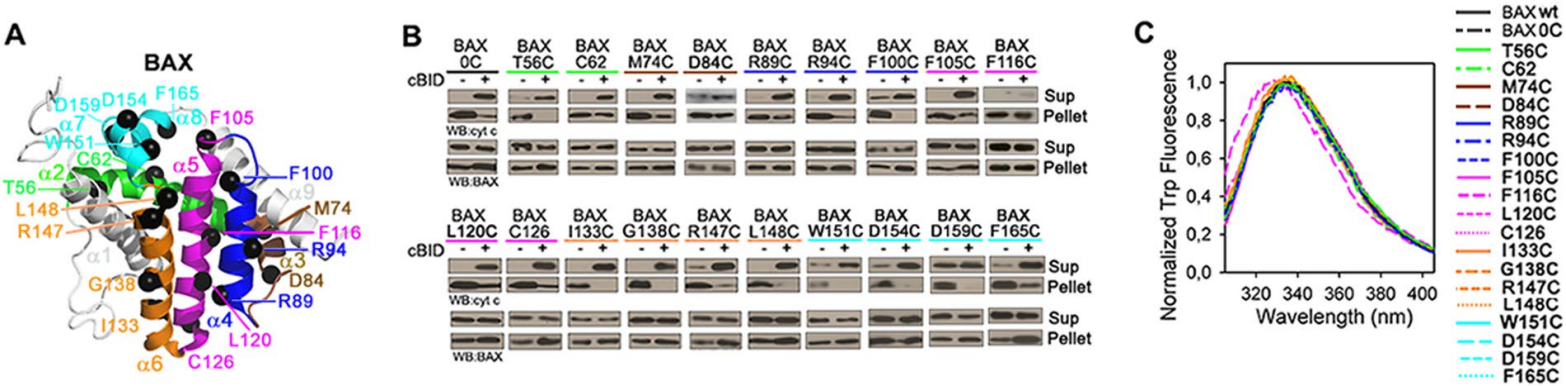
Characterization of BAX mutants. (**A**) Inactive BAX structure (PDB 1F6) showing Cys mutation sites (black spheres). **(B)** Cyt-c-releasing and mitochondrial-localizing activities of BAX proteins. Data representative of at least two independendent experiments. **(C)** Trp fluorescence spectra of BAX proteins. Spectra representative of three independent experiments.

Using as a template Cysteine (Cys)-less BAX (designated as BAX 0C), we generated a set of nineteen recombinant BAX monocysteine mutants to map the membrane topology and role in pore formation of specific BAX regions. The three-dimensional NMR solution structure of inactive, monomeric BAX is shown in Fig. 1A, with residues mutated to Cys highlighted as black spheres and BAX helical segments colored according to the following scheme: BAX α2, green; BAX α3,brown; BAX α4, blue; BAX α5, pink; BAX α6, orange; and BAX α7-α8, cyan.

We first assessed the functional integrity of monocysteine BAX variants by examining their capacities to release mitochondrial cyt c with or without the BH3-only activator ligand, cBID. As observed with BAX wild-type (BAX wt) and BAX 0 C, most monocysteine BAX mutants displayed minimal cyt c releasing activity in the absence of cBID, and near complete cyt c release in its presence (Fig. 1B, and Supplementary Fig. S1). The exceptions were the “autoactive” BAX D159C variant showing prominent cyt c release without cBID, and the “inactive” BAX D84C and BAX F116C variants which only showed limited cyt c release with cBID. Further immunoblotting analyses indicated that most cBID-activated BAX variants targeted to mitochondria similarly to BAX 0 C, although the latter assay proved less sensitive than that of cyt c release (Fig. 1B).

To test whether Cys mutations affect the structural integrity of the protein, we first compared the net wavelength of tryptophan (Trp) maximum emission (λ_max_) for the different proteins. As shown in Fig. 1C, Trp λ_max_ values for BAX wt, BAX 0 C, and all monocysteine BAX mutants were very similar. The only exception was BAX F116C mutant which showed a 6 nm blue-shift in Trp λ_max_, probably because the Cys residue in this variant is localized at the very core of the BAX molecule (Fig. 1A). To further examine the effect of Cys substitutions on BAX structure we performed Differential Scanning Fluorimetry (DSF) experiments. The majority of BAX monocysteine mutants present DSF spectra very similar to that of BAX wt, with the differences between the melting temperatures (T_m_) of most BAX variants and that of BAX wt being less than 5 °C (Supplementary Fig. S1). The exceptions were BAX W151C and BAX D159C mutants. Of note, despite the T_m_ of BAX W151C is ≈5 °C lower than that of BAX, this mutant behaved as the native protein in the cyt c release assay.

Based on this collective set of results, we concluded that most monocysteine BAX variants maintain the structural and functional integrity of the parent molecule. The exceptions were BAX D84C, BAX F116C, and BAX D159C variants, which were not examined further.

### Assessing the active structure of BAX at the membrane level by fluorescence mapping

Fluorescence spectroscopy coupled to site-directed fluorescence labeling is a powerful methodology to study conformations of membrane-associated proteins. In this experimental approach, a fluorescent dye is covalently attached to the sulfhidryl group of a protein containing a single Cys residue positioned at a strategic site. NBD was chosen as the fluorescent probe in this study because it is a relatively small and uncharged dye, and its spectral properties are dramatically different in aqueous and nonaqueous environments^28,34,35^. For example, when NBD moves from a polar enviroment (i.e. the hydrophilic surface of a protein) to a nonpolar environment (i.e. the hydrophobic interior of the membrane or a protein), its fluorescence emission intensity (F) increases while its λ_max_ shifts to the blue.

**Figure 2:**
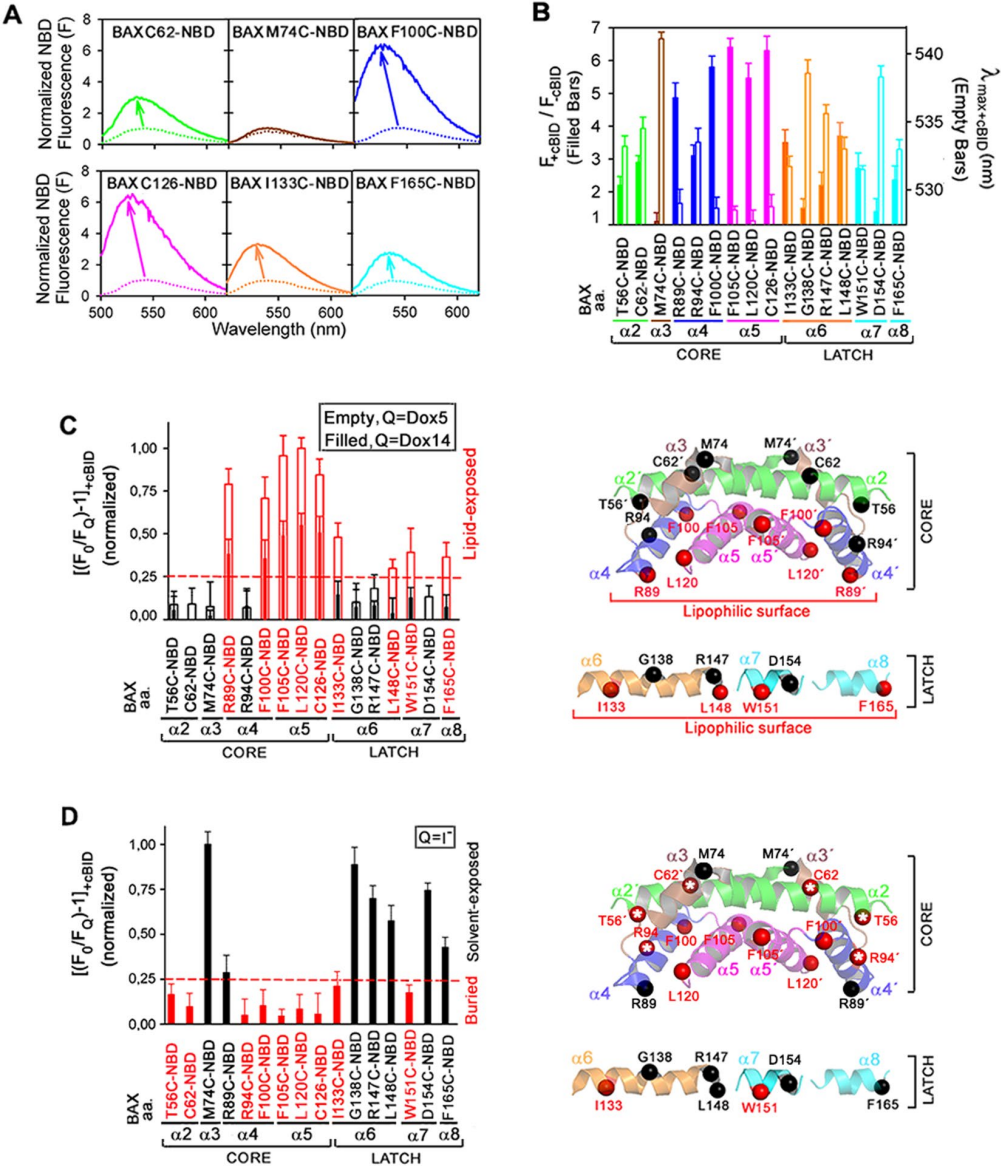
Fluorescence mapping of membrane active BAX topology. (**A**) Representative **e**mission spectra of NBD-BAX variants with (continuous lines) or without (dotted lines) cBID. (**B**) Filled bars: NBD intensity ratios for cBID-activated to inactive NBD-BAX variants. Empty bars: NBD λ_max_ for cBID-activated NBD-BAX variants. **(C)** Left: Dox-quenching ratios for cBID-activated NBD-BAX variants. Right: Structures of dimeric BAX core α2-α5 helices (extracted from PDB 4BDU) and BAX latch α6-α8 helices (extracted from PDB 1F16) depicting Dox5-exposed (red spheres) and -unexposed (black spheres) residues. **(D)** Left: I^−^-quenching ratios for cBID-activated NBD-BAX variants. Right: BAX structures depicting solvent-exposed (black spheres) and -unexposed (red spheres) residues. Throughout Figure, graphs show mean ± S.E.M. (n ≥ 3 technical replicates).

Numerous studies have shown that BAX activation and action during apoptosis can be accurately reproduced in a minimalist cell-free assay consisting of recombinant BAX and cBID proteins, together with MOM-like liposomes^11,15,16,22,23,25,28^. Figure 2A shows typical emission scans for selected NBD-labelled BAX mutants in the presence of MOM-like liposomes with or without cBID treatment (continuous and dotted lines, respectively). Upon treatment with cBID, most mutants exhibited notable increases in F and blue-shifts in λ_max_ indicating transfer of NBD probes to more hydrophobic environments (Fig. 2A, arrows). Quantitative assessment of cBID-elicited NBD-BAX changes in F (Fig. 2B, filled bars) and λ_max_ (Fig. 2B, empty bars) suggests that BAX residues can be grouped in the following three broad categories: (1) sites which go through prominent changes in F and are placed in hydrophobic microenvironments as assessed by their low λ_max_ values; i.e., R89 and F100 in BAX α4, and F105, L120, and C126 in BAX α5; (2) sites which undergo small to negligible changes in F and localize to hydrophilic microenvironments according to their high λ_max_ values; i.e., M74 in BAX α3, G138 in BAX α6, and D154 in BAX α7; and (3) sites which experience rather similar changes in F ranging from moderate to notable and localize to microenvironments of middle polarity judging by their intermediate λ_max_ values; i.e., T56 and C62 in BAX α2, R94 in BAX α4, I133, R147, and L148 in BAX α6, W151 in BAX α7, and F165 in BAX α8.

Although the spectroscopic approach described above provides valuable information, alone it does not allow distinguishing whether an NBD probe shifts to a nonpolar environment by becoming exposed to the hydrophobic interior or the interface of the lipid bilayer, or by moving into a hydrophobic site within a proteinaceous structure. To further evaluate the membrane topography of each NBD probe in active BAX variants we performed a series of collisional quenching experiments. We first used the lipophilic quenching agents 1-palmitoyl-2-stearoyl-5-doxyl-sn-glycero-3-phosphocholine (Dox5) and 1-palmitoyl-2-stearoyl-14-doxyl-sn-glycero-3-phosphocholine (Dox14), two phospholipid derivatives with a NO group localized within the bilayer hydrophobic phase, close to the glycerol backbone and the bilayer midplane, respectively^36^. The left Panel in Fig. 2C is a histogram showing the extent of quenching by doxylated lipids for the set of monocysteine BAX mutants incubated with MOM-like liposomes and cBID. As can be seen, NBD probes attached to R89, F100, F105, L120, and C126 sites in cBID-activated BAX were substantially quenched by both Dox5 and Dox14, with the former lipid eliciting stronger quenching than the latter one. Thus, this set of residues localized in the BAX core α4-α5 region are placed within the hydrocarbon phase of the lipid bilayer, but without reaching the bilayer midplane. By contrast, NBD attached to other sites in the BAX core domain (T56, C62, M74, and R94) and a group of sites localized in the BAX latch domain (G138, R147, and D154) showed negligible quenching by either Dox5 or Dox14 indicating these residues do not penetrate into the hydrocarbon phase of the lipid bilayer when BAX acquires its active conformation. Lastly, a set of sites localized in the BAX latch domain (I133, L148, W151, and F165) displayed considerable quenching by Dox5 but minimal quenching by Dox14, suggesting these residues are peripherally attached to the membrane surface in cBID-activated BAX.

Next, the Dox5 quenching results for sites in the BAX core domain were mapped into the BAX core BH3-in-groove dimer crystal structure^5^ (Fig. 2C, right). It is readily apparent that NBD sites showing strong quenching by Dox5 localize to the largely hydrophobic “bottom” part of the dimeric BAX core crystal structure expected to provide a lipophilic surface in the molecule (red spheres), while NBD sites showing weak quenching by Dox5 are distributed along regions of the dimeric BAX core crystal structure expected not to interact with membrane lipids (black spheres). Thus, Dox5 quenching results obtained with cBID-activated BAX in MOM-like liposomes fit nicely into this crystallographic BAX core dimer structure. On the other hand, mapping the Dox5 quenching results obtained for sites in the BAX latch domain into structural models for BAX α6, α7 and α8 helices reveals a potential lipophilic surface comprising the most hydrophobic faces of each one of these three helices. It should be emphasized here that despite our Dox-quenching experiments identified multiple “lipid-exposed” residues at different positions along BAX core and latch helices, none of these BAX sites showed higher quenching levels by Dox14 relative to Dox5. This supports–but does not prove- the idea that BAX core and latch helices do not adopt a TM orientation when BAX acquires its active conformation^5,11,20^.

We next examined the same cBID-activated NBD-BAX mutants for quenching by the hydrophilic quencher, Iodide (I^−^) (Fig. 2D, left). NBD attached to sites R89, F100, F105, L120, and C126 in BAX α4-α5 displayed modest to minimal quenching by I^−^, consistent with Dox-quenching results indicating that all these residues of the BAX core domain are buried within the hydrophobic membrane interior in cBID-activated BAX (Fig. 2C, left). NBD attached to sites T56, C62, and R94 in the BAX core domain also displayed weak quenching by I^−^ (Fig. 2D, left), which together with their minimal quenching by doxylated lipids (Fig. 2C, left), strongly suggests that these three residues are hidden within a hydrophobic proteinaceous structure in active BAX. By contrast, NBD attached to M74 site in the BAX core domain and to multiple sites along the BAX latch domain (G138, R147, L148, D154, and F165) showed prominent quenching by I^−^. Thus, all these residues are predominantly exposed to aqueous solution when BAX acquires its active conformation. Of note, a general, although not complete, coherence was found among BAX latch residues regarding their relative I^−^- and Dox5-quenching levels. For instance, G138, R147, and D154 residues showed high I^−^- quenching levels (Fig. 2D, left) and low Dox5-quenching levels (Fig. 2C, left), L148 and F165 displayed somewhat lower I^−^-quenching levels and somewhat higher Dox5-quenching levels, and I133 and W151 showed low I^−^-quenching levels and considerable Dox5-quenching levels.

Mapping I^−^ quenching results for sites in the BAX core domain into the BAX core BH3-in-groove dimer crystal structure also revealed a general agreement between experimental results and the distribution of BAX residues according to this structural model, as follows (Fig. 2D, right). First, all residues in the BAX α4-α5 region expected to be hidden at the “bottom” lipophilic surface of the dimeric BAX core structure scored as “buried” by the I^−^quenching approach. Despite R89 in the putative lipophilic surface of BAX α4 scored as “solvent-exposed”, this residue displayed the smallest I^−^ quenching levels among all “solvent-exposed” residues in cBID-activated BAX (Fig. 2D, left). Second, residue M74 in BAX α3 that strongly scored as “solvent-exposed” by I^−^ quenching method localizes to a surface-exposed region at the “top” of the dimeric BAX core crystal structure. Third, residues T56 and C62 in BAX α2 and R94 in BAX α4 scoring as “buried” by the I^−^ quenching approach localize to the protein:protein interface between the two BAX monomers in the dimeric BAX core crystal structure (red spheres with white stars). It should be mentioned that although our fluorescence mapping assays do not directly measure BAX dimerization, previous cysteine cross-linking data indicated that T56, C62, and R94 residues are at least partially buried within a BH3-in-groove dimeric BAX conformer at the MOM level^8,10^. On the other hand, the mapping of I^−^ quenching results for sites in the BAX latch domain into structural models for BAX α6, α7 and α8 helices sustains the view that the entire latch region of the activated BAX molecule adopts a peripheral disposition at the membrane surface showing extensive exposure to the aqueous environment. Despite some inconsistencies, relative I^−^ quenching levels of different BAX latch residues generally support the idea that the BAX latch domain displays a lipophilic surface encompassing the most hydrophobic faces of its component helices.

Overall, fluorescence mapping of active BAX topology in MOM-like membranes indicates that the BAX core domain adopts a BH3-in-groove dimeric structure presenting a lipophilic surface in the BAX α4-α5 region, while the BAX latch domain provides another lipophilic surface along one side of its constituent α6-α8 helices. In addition, the combined results also reveal that the BAX core α4-α5 helices penetrate deeper into the hydrocarbon region of the membrane lipid bilayer than the BAX latch α6-α8 helices.

### BCLXL blocks membrane insertion of BAX core, not latch domain

**Figure 3:**
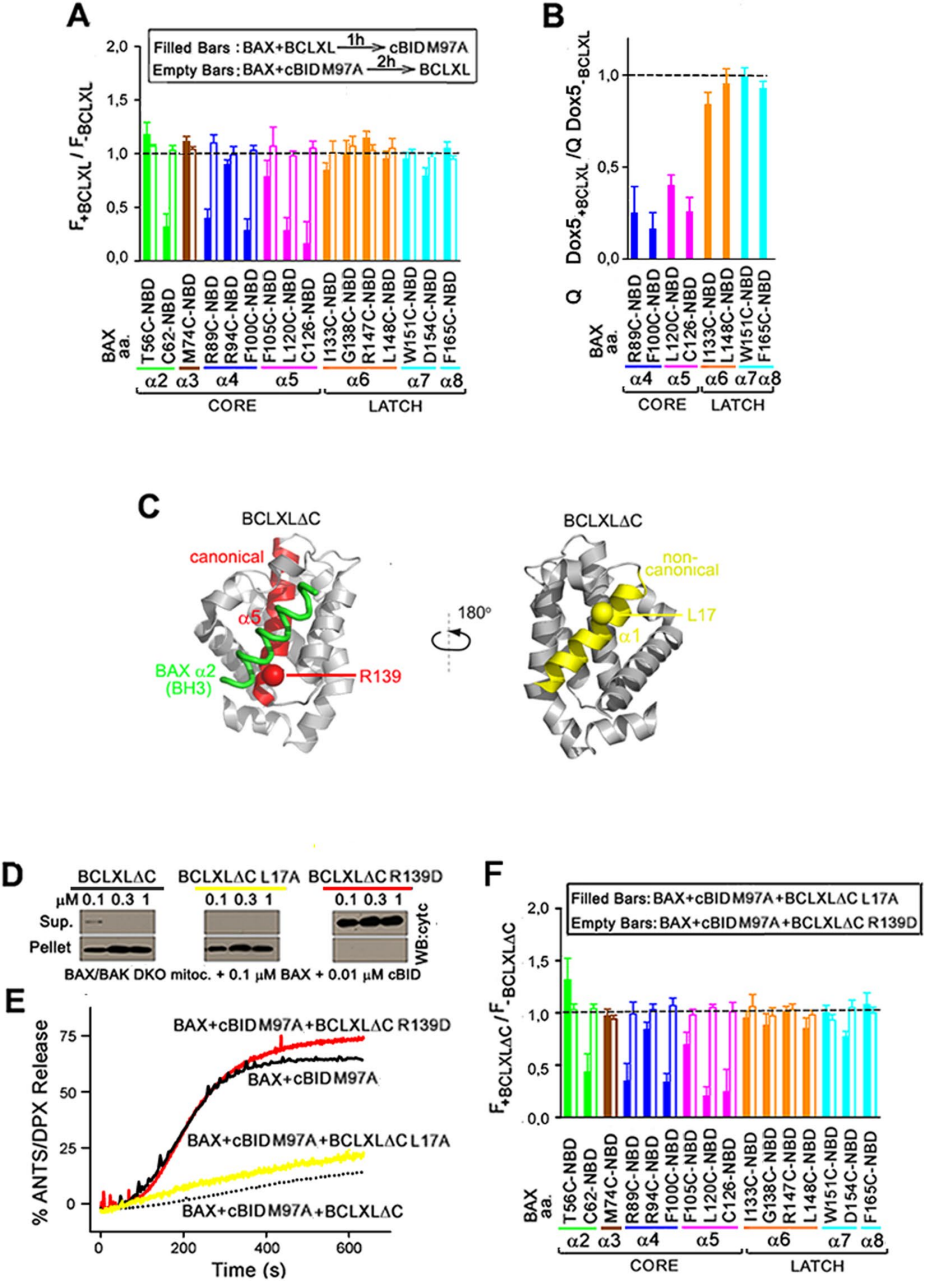
Mode of BCLXL inhibition. (**A)** Intensity ratios of NBD-BAX (BAX) variants treated with cBID M97A plus BCLXL (F_+BCLXL_) to those treated with cBID M97A alone (F_−BCLXL_). NBD-BAX was treated with BCLXL 1 h before (filled bars) or 2 h after (empty bars) cBID M97A addition. (**B**) Dox5-quenching ratios for NBD-BAX variants treated with cBID M97A plus BCLXL (QDox5_+BCLXL_) to those treated with cBID M97A alone (QDox5_−BCLXL_). **(C**) Representation of BCLXLΔC:BAX BH3 complex (PDB: 3pl7) highlighting critical helices and residues at BCLXLΔC canonical (red) and non-canonical (yellow) surfaces. **(D**) Mitochondrial cyt c release by BAX, cBID M97A, and BCLXLΔC. Assays repeated two times using two independent preparations of mitochondria and proteins with identical results. **(E)** ANTS/DPX release kinetics elicited by BAX, cBID M97A, and BCLXLΔC in MOM-like LUVs. Kinetics representative of three independent experiments. **(F)** As in Panel A. Throughout Figure, graphs show mean ± S.E.M. (n ≥ 3 technical replicates).

Next, we analyzed the effect of antiapoptotic BCLXL on BAX membrane topology using fluorescence mapping. For these experiments we used the cBID M97A mutant which displays negligible binding to BCLXL but preserves intact BAX activation capacity^32^. We also considered the ongoing debate on whether antiapoptotic proteins neutralize BAX exclusively through canonical BH3-in-groove heterodimeric interactions, or also via additional non-canonical protein-protein binding interactions^16,29–33,37^. In the former case, BCLXL is expected to exert its inhibitory action only before cBID had triggered the BAX BH3-in-groove dimerization process, while in the latter scenario BCLXL is predicted to remain at least partially active even after BAX has become previously dimerized by cBID. Interestingly, adding BCLXL to BAX before cBID M97A inhibited the fluorescence increase of NBD attached to multiple sites in BAX α2-α5, but not α6-α8 helices, suggesting that under these conditions BCLXL selectively inhibits membrane insertion of the BAX core, but not latch domain (Fig. 3A, filled Bars). By contrast, when BCLXL was added after cBID M97A had activated BAX, insignificant changes were observed in the NBD fluorescence of all BAX variants examined (Fig. 3A, empty bars).

To directly test whether BCLXL selectively blocks membrane insertion of BAX core domain, we assessed the effect of BCLXL on Dox5-mediated quenching of different NBD-BAX variants. Indeed, BCLXL markedly inhibited the NBD quenching elicited by Dox5 at multiple sites in the BAX core (BAX R89C, BAX F100C, BAX L120C, and BAX C126), but not latch domain (BAX I133C, BAX L148C, BAX W151C, and BAX F165C) (Fig. 3B).

To try to further discriminate between canonical and non-canonical mechanisms of BCLXL-mediated BAX inhibition, we used the BCLXLΔC R139D and BCLXLΔC L17A variants expected to disrupt canonical and non-canonical BCLXL:BAX binding interfaces, respectively (Fig. 3C)^2,37^. The canonical BCLXLΔC R139D mutant completely lost the ability of native BCLXLΔC to inhibit cBID-mediated BAX activation as determined by measurements of mitochondrial cyt c release (Fig. 3D), vesicular ANTS/DPX release (Fig. 3E), and NBD-BAX fluorescence mapping (Fig. 3F). In contrast, the BCL2-like non-canonical BCLXLΔC L17A mutant preserved all these inhibitory activities displayed by the parent protein (Fig. 3D–F).

Thus, we concluded that antiapoptotic BCLXL inhibits both membrane insertion of BAX core domain and BAX apoptotic pore formation via canonical BH3-in-groove interactions.

### PEGylation of multiple individual sites in the BAX core, but not latch domain, blocks BAX apoptotic pore formation

Next, we used site-specific PEGylation^38^ to analyze the specific contribution of invidual BAX core and latch residues to BAX apoptotic pore formation. To this aim, we modified the different BAX monocysteine mutants with the small hydrophilic reagent methoxy PEG maleimide of 550 Da (PEG05k) to yield a set of mono-PEGylated BAX variants, and then compared the membrane-permeabilizing activity of each BAX mutant with or without site-specific PEGylation. The rationale behind this experimental approach is that conjugation of a hydrophilic PEG05k molecule at a specific site in BAX should obstruct the localization of that particular BAX residue to the hydrophobic interior of the membrane or a BAX oligomerization interface, thereby potentially inhibiting BAX-induced membrane permeabilization.

**Figure 4:**
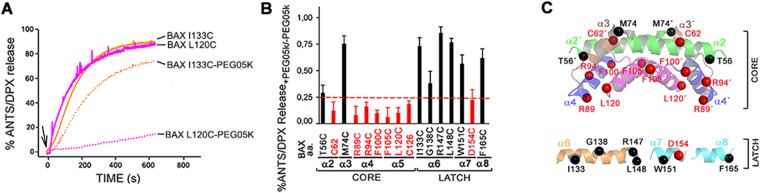
Contribution of BAX core and latch residues to membrane permeabilization. **(A)** Representative ANTS/DPX release kinetics elicited by cBID-activated BAX variants conjugated or unconjugated with PEG05k. Arrow: cBID. **(B)** Ratios of ANTS/DPX release by BAX variants conjugated with PEG05k to BAX unconjugated with PEG05k. Data show mean ± S.E.M. (n ≥ 3 technical replicates). **(C)** BAX structures depicting residues strongly (red spheres) or weakly (black spheres) inhibiting BAX pore formation upon PEG05k conjugation.

BAX-induced liposome permeabilization was inhibited at different degrees depending on the site of PEGylation (Fig. 4A,B). Basically, PEGylation of all sites at the BAX core domain potently inhibited BAX permeabilizing activity, except for BAX M74 site. By contrast, PEGylation of all sites at the BAX latch domain had a generally weaker effect on BAX permeabilizing activity, except for BAX D154 site.

The relative impact of site-specific BAX PEGylation on BAX-induced membrane permeabilization was mapped into BAX structural models (Fig. 4C, Right). These representations, together with those shown in Fig. 2, illustrate that (i) BAX sites where PEGylation strongly inhibits BAX-induced membrane permeabilization comprise residues at the BAX core domain implicated in BAX BH3-in-groove dimerization (C62, R94) and BAX α4-α5 membrane insertion (R89, F100, F105, L120, C126); whereas (ii) BAX sites where PEGylation weakly inhibits BAX-induced permeabilization essentially encompass the solvent-exposed BAX core M74 residue together with multiple residues localized at the peripherally membrane-attached BAX latch α6-α8 region (I133, G138, R147, L148, W151, and F165).

### BAX core α5 peptide displays membrane activitites that are absent in BAX latch α6 and α7-α8 peptides

**Figure 5:**
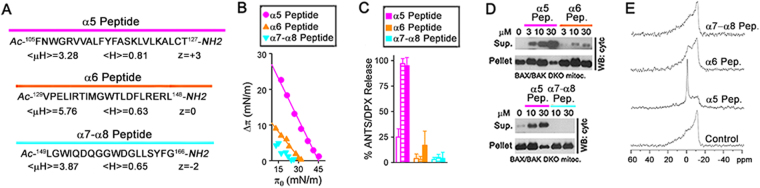
Membrane activities of BAX-derived peptides. (**A**) Main biophysical characteristics of BAX-derived peptides. **(B)** Effect of BAX-derived peptides on lipid monolayer surface pressure. Data representative of four independent experiments. **(C)** Extents of ANTS/DPX release elicited by BAX-derived peptides at 0.1 μM (empty bars), 1 μM (stripped bars), and 5 μM (filled bars). Data show mean ± S.E.M. (n ≥ 3 technical replicates). (**D**) Cyt c release by BAX-derived peptides. Assays repeated three times with similar results. (**E**) Effect of BAX-derived peptides on membrane lipid bilayer structure assessed by 31 P NMR.

As an additional approach to try determining the role of BAX core and latch helices in BAX apoptotic pore formation, we decided to examine different membrane activities of synthetic peptides representing BAX α5, α6, and α7-α8 regions. We first determined the main biophysical properties of BAX α5, α6, and α7-α8 regions using MPEx and Heliquest^39,40^. The BAX core α5 helix showed higher mean hydrophobicity (<H>), lower amphipathicity (<μH>), and more positive net charge (z) than the BAX latch α6 and α7-α8 helices (Fig. 5A).

Next, the capacity of BAX-derived peptides to penetrate into MOM-like lipid monolayers was assessed (Fig. 5B). For BAX α5 and BAX α6 peptides, the change in lipid monolayer surface pressure (Δp) upon peptide addition decreased linearly as a function of increasing initial surface pressure (π_0_), giving critical surface pressure (π_c_) values of 34.8 mN/m and 25.6 mN/m, respectively. Considering that typical π_c_ values for lipid bilayer membranes are in the range of 25–30 mN/m^41^, these data suggest that the BAX α5 peptide displays a superior capacity to penetrate into the MOM lipid bilayer compared to the BAX α6 peptide. In parallel, we compared the membrane-permeabilizing ability of BAX-derived peptides. As shown in Fig. 5C, the BAX α5 peptide induced ANTS/DPX release from MOM-like LUV in a dose-dependent manner, while the BAX α6 and BAX α7-α8 peptides were much less active in this experimental system. Similarly, the BAX α5 peptide induced a dose-dependent depletion of cyt c in BAX/BAK DKO mitochondria, whereas the BAX α6 and BAX α7-α8 peptides virtually did not release any mitochondrial cyt c at any concentration tested (Fig. 5D).

31P NMR studies were also conducted to directly assess whether these peptides disrupt the membrane lipid bilayer structure. The 31P NMR spectrum of MOM-like liposomes showed the high-field peak and low-field shoulder typical of a planar bilayer arrangement of membrane lipids (Fig. 5E). Addition of the BAX α5 peptide to MOM-like liposomes led to a profound change in the shape of the 31P NMR spectrum: the bilayer-type signal markedly decreased while a prominent peak appeared around the chemical shift position of phospholipids experiencing isotropic motion, which is typical for highly curved non-bilayer type lipid dispositions. By contrast, the BAX α6 and BAX α7-α8 peptides did not significantly alter the 31 P NMR spectrum of MOM-like liposomes.

Collectively, these results revealed that the BAX core α5 helix possesses the capacity to insert into the MOM lipid matrix, destabilize the MOM lipid bilayer structure, and breach the MOM permeability barrier, while the BAX latch α6-α8 helices lack such intrinsic membrane activities.

### Computational simulations reveal dissimilar membrane interaction modes for the BAX core α5 helix, the BAX latch α6-α8 helices, and the BAX C-terminal α9 helix

Finally, we performed coarse-grained Monte Carlo (MC) simulations of peptides in association with MOM-like lipid bilayer membranes using the MCPep web server^42^. Although this computational model captures only certain characteristics of the complex peptide-lipid system, it allows obtaining quantitative information of thermodynamic parameters reflecting the mode of peptide-membrane interaction; in particular, the peptide´s membrane-association free energy (ΔG_total_), favored membrane orientation (Tilt), and preferred membrane penetration depth (Z_center_). Moreover, the MC simulation model has been previously tested for a variety of peptide and protein fragments in membrane environments, and reproduced available empirical data and results obtained with explicit molecular dynamics simulations with reasonable success^42–44^.

**Figure 6:**
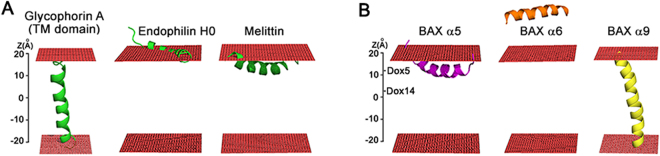
Peptide-membrane association modes assessed by MC simulations. **(A)** Example peptides; **(B)** BAX-derived peptides. Red rectangles represent phospholipid headgroups.

We first examined three experimentally well-studied case examples in this computational system (Fig. 6A): (1) the prototypical TM domain of glycophorin A^45^; (2) the N-terminal H0 helix of endophilin A1 localizing at the level of the phospholipid phosphate groups^46^; and (3) melittin, a potent pore-forming and bilayer-destabilizing cytolitic peptide that localizes at the upper region of the hydrocarbon phase of the lipid bilayer^47^. Indeed, for each one of these example cases analyzed, the MCPep simulation successfully reproduced the expected peptide-membrane interaction mode (Fig. 6A, and Supplementary Table S1).

We next examined the membrane-interaction modes of BAX α5, α6, α7-α8, and α9 peptides by MCPep (Fig. 6B, and Supplementary Table S1). Remarkably, the BAX core α5 peptide displayed a membrane-interaction mode very similar to that of the melittin peptide, by localizing into the sub-surface region of the membrane with a membrane-association free energy of −26.1 kT, its geometrical center at an average distance of 18.1 Å from the membrane midplane, and its principal axis nearly parallel to the membrane surface. By contrast, the BAX latch α6 and α7-α8 peptides interacted very weakly with the membrane (ΔG_total_ < 5 kT), and for the most part, remained in the aqueous phase (Z_center_ > 30 Å). Lastly, the most energetically favored disposition for the BAX C-terminal α9 peptide was the TM orientation.

Thus, the dissimilar membrane interaction modes of the BAX core α5 peptide compared to the BAX latch α6 and α7-α8 peptides disclosed by MCPep simulations concur with experimental results showing that only the former peptide possesses membrane-inserting and bilayer-destabilizing activities (Fig. 5). MCPep computational results also qualitatively agree with fluorescence mapping studies of active BAX in MOM-like LUVs showing that the BAX core α5 helix inserts deeper into the membrane lipid bilayer than BAX latch α6-α8 helices (Fig. 2).

## Discussion

How BCL2 family proteins modulate apoptosis through MOM permeability changes has been intensively studied during the last two decades^1,2,4,14,27,30^. However, a comprehensive view of this fundamental process regulating cell fate is still lacking. Here, using a variety of biophysical and biochemical techniques applied to minimalist *in vitro* reconstituted systems, we provide new insight into how BAX and BCLXL regulate the formation of mitochondrial apoptotic pores through specific protein:protein and protein:lipid interactions.

Our work adds significantly to a growing number of studies indicating that the BAX BH3-into-groove dimerization process plays a fundamental role in BAX-elicited apoptotic pore formation^5,8,10,11,20^. Not only did we show that the BAX BH3-in-groove dimeric conformation persists in the fully active conformation of BAX rather than merely being an intermediate in the molecular pathway for BAX activation (Fig. 2); we also revealed that PEGylation of multiple individual BAX core residues implicated in BAX BH3-in-groove dimerization effectively blocks the BAX pore-forming activity (Fig. 4). By contrast, our studies do not support the so-called BAX α2α3α4 dimeric structure for fully active BAX, although we cannot discard that BAX may transiently adopt this alternative dimeric structure at early stages of its functional activation pathway^8^. Concerning higher order BAX oligomerization, site-specific fluorescence mapping and PEGylation results are consistent with the view that stable BAX BH3-in-groove dimers can grow into more dynamic BAX multimeric species through multiple BAX interdimer interfaces localized throughout BAX core, latch, and C-terminal domains^7–14,18^. In this scenario, the high mobility of such BAX interdimer interfaces would preclude their detection by the steady-state fluorescence analyses used here, while PEGylation of a single BAX interdimer interface would not be sufficient to efficiently block BAX multimerization and pore formation.

Another ongoing debate in the BCL2 research field pertains to the precise protein:protein interaction mechanisms through which BCL2-type proteins inhibit BAX-type proteins during apoptosis^26–33,37^. According to canonical models, antiapoptotic proteins neutralize proapoptotic partners via heterodimeric BH3-in-groove complexes that in principle, should be formed before BAX BH3-in-groove homodimers had been assembled. On the other hand, non-canonical models postulate that antiapoptotic proteins can use binding interfaces other than their canonical groove to form inactive complexes with BAX-type proteins, conceptually even dissasembling preformed BAX complexes. In this regard, the differential effects exerted by the sequential addition of BCLXL and cBID M97A on BAX membrane topology (Fig. 3A) together with the opposite effects exerted by canonical and non-canonical BCLXLΔC mutants on BAX membrane activities (Fig. 3D–F) indicate that BCLXL inhibits BAX proapoptotic action exclusively by sequestering the BAX BH3 domain into its canonical groove. Nevertheless, our results are not incompatible at all with the possibility that non-canonical BCLXL:BAX interactions may regulate normal cell physiology processes^48^.

Another important finding of our studies is that BAX apoptotic pore formation is driven by lipid interactions established by BAX core α4-α5 helices, but not BAX latch α6-α8 helices, despite both regions of BAX associate with the membrane lipid bilayer when the protein acquires its active conformation. Experimental and computational data indicate that the primary origin of this dissimilar behavior of BAX core and latch helices is their differential membrane penetration degrees: BAX α4-α5 localize to the upper region of the hydrocarbon core of the lipid bilayer, whereas BAX α6-α8 localize to a more superficial region of the membrane interface. This is in line with current knowledge on the membrane penetration-depth of pore-forming helical peptides such as melittin, which in that way produce the optimal surface tension and curvature stress within the membrane required to destabilize its lipid bilayer structure and open a proteolipidic pore therein^47,49,50^. The finding that BCLXL blocks insertion of BAX core α4-α5 helices into the membrane without significantly affecting membrane insertion of BAX latch α6-α8 helices further supports that the former process is a more important contributor of BAX pore formation than the latter one.

Our results prompt to reconsider certain assumptions made in recent models proposed to explain proteolipidic pore formation by BAX-type proteins. Specifically, the clamp model postulates that insertion of the BAX latch α6-α8 region into the MOM lipid bilayer is a key determinant of BAX proteolipidic pore formation^11^. However, the degree of membrane insertion of BAX α6-α8 helices and the contribution of the BAX latch region to BAX pore-forming activity were not explicitely examined in that work^11^. Similarly, the in-plane model proposes that BAX proteolipidic pore formation is driven by shallow membrane insertion of multiple BAX helices, potentially including all helices belonging to the BAX latch domain^20^. However, in that study the topological analyses of the BAX latch domain were limited to part of the BAX α6 helix (up to BAX L144 residue)^20^. Moreover, BAX membrane topology was assessed at the mitochondrial level using a chemical labelling technique providing lower spatial resolution than the fluorescence spectroscopy approaches applied here to BAX integrated in MOM-like liposomal membranes. Nevertheless, our results are not necessarily incompatible with the proposal of the in-plane model stating that the BAX latch domain stabilizes a nascent BAX proteolipidic pore by sliding into the pore lumen in such a manner that decreases its line tension^20^. The intrinsic curvature of the dimeric BAX core domain may also contribute to enrichment of BAX molecules at the pore edge^25^, thereby reducing pore line tension and stabilizing the open pore state as hypothesized in the clamp model^11,17^.

In conclusion, our study provides new structural and mechanistic information into how BAX forms lethal mitochondrial pores. We have described experimental approaches that can precisely monitor BAX membrane conformations and activities which may impact on the development of therapeutics that target this crucial proapoptotic protein, and could potentially be used with other BCL2 family members as well.

## Materials and Methods

### Chemicals and reagents

Phosphatidylcholine (PC), phospatidylethanolamine (PE), phosphatidylinositol (PI), cardiolipin (CL), and doxylated lipids (Dox5 and Dox14) were from Avanti Polar Lipids (Alabaster, AL, USA). *N*,*N*′-Dimethyl-*N*-(Iodoacetyl)-*N*′-(7-Nitrobenz-2-Oxa-1,3-Diazol-4-yl)Ethylenediamine (NBD), 1, 3, 6, aminonaphtalene-tri-sulfonate (ANTS) and p-xilene-bis-dipicolyinicacis (DPX) were purchased from Molecular Probes (Eugene, OR, USA). Methoxy PEG maleimide of 550 Da average molecular weight (PEG05k) was obtained from Nanocs (New York, NY, USA). Synthetic peptides (>90% purity) were purchased from Biomatik (Wilmigton, DL, USA). All other reagents were from Sigma (St. Louis, MO, USA).

### Liposome preparation

MOM-like lipid mixtures (PC/PE/PI/CL 50/35/10/15, mol/mol) were co-dissolved in chloroform/methanol (2:1), and organic solvents were removed by incubation under vacuum for 2 h. Dry lipid films were resuspended in 100 mM KCl, 10 mM Hepes, pH 7.0 1 mM EDTA (KHE buffer), except in experiments were 20 mM KCl, 10 mM Hepes pH 7.0, 1 mM EDTA, 12.5 mM ANTS and 45 mM DPX was used. Liposomes were then subjected to 10 freeze/thaw cycles, and subsequently extruded 10 times through two polycarbonate membranes of 0.2-μm pore size (Nucleopore, San Diego, CA) to obtain large unilamellar vesicles (LUVs).

### Purification and labeling of recombinant BCL2 family proteins

Mutant DNAs were generated by PCR-based mutagenesis using the Quickchange mutagenesis kit (Stratagene, San Diego, CA, USA) or purchased at GenTech (Montreal, Canada). All constructs were verified by sequencing. Full-length human BAX (designated as BAX wt), BAX with two native cysteines substituted by serine (BAX C62S, C126S, designated as BAX 0C), BAX mutants with a single cysteine, and full-length human BCLXL (designated as BCLXL), were all expressed in *Escherichia coli* BL21 (DE3) using the pTYB1 vector (New England Biolabs, Ipswich, MA). Cells were induced with 0.5-1 mM isopropyl-1-thio-β-D-galactopiranoside overnight at 18 °C. The harvested cells were lysed at 4 °C with a homogenizer (EmulsiFlex C5, Avestin, Ottawa, ON, Canada) in 500 mM NaCl, 10 mM Tris pH 8.0, 1 mM EDTA, 5 mM MgCl_2_, 10% glycerol, 1 mg/ml lysozyme, 2.5 ug/ml DNase I, and cOmplete protease inhibitor cocktail tablets (Roche, Basel, Switzerland). BAX and BCLXL proteins were isolated from the supernatant by chitin affinity chromatography according to the protocol from the vendor (New England Biolabs, Ipswich, MA), and further purified on a Superdex-75 size-exclusion column (GE Healthcare, Uppsala, Sweden). Purified BAX and BCLXL fractions were concentrated using Amicon spin filters, and dialyzed in KHE buffer (100 mM KCl, 10 mM Hepes, pH 7.5, 1 mM EDTA) supplemented with 10% glycerol and 1 mM tris(2-carboxyethyl)phosphine (TCEP). cBID and BCLXLΔC (BCLXL lacking the C-terminal 24 aminoacids) were expressed and purified as described earlier^23,51^. All protein preparations were >90% pure as assessed by sodium dodecyl sulfate polyacrylamide gel electrophoresis (SDS-PAGE) and Coomassie-blue staining. In a typical protein labeling reaction, NBD or PEG05k was incubated with a monocysteine BAX variant at a 10:1 molar ratio overnight at 4 °C, followed by elution over a PD-10 column in KHE supplemented with 10% glycerol and 1 mM TCEP.

### Cyt c release and BAX immunodetection assays

BAX^−/−^/BAK^−/−^DKO mouse embryonic fibroblasts (MEFs) were harvested by scrapping, and homogenized with a glass-Teflon Potter-Elvehjem homogenizer in mitochondrial isolation buffer (210 mM mannitol, 70 mM sucrose, 10 mM Hepes (pH 7.5), 1 mM EDTA, and protease inhibitors). After removing heavy membrane fractions by two consecutive centrifugations at 700 *g* for 10 min at 4 °C, mitochondria-enriched fractions were pelleted by centrifuging the resultant supernatant at 14000 *g* for 10 min at 4 °C. Mitochondria (50 μg total protein) were incubated with recombinant BAX variants (100 nM) with or without cBID (10 nM) in 125 mM KCl, 5 mM KH_2_PO_4_, 2 mM MgCl_2_, 1 mM DTT, and 10 mM HEPES-KOH, pH 7.2, for 30 min at 30 °C. Samples were then centrifuged at 14000 *g* for 10 min, and supernatant and pellet fractions were subjected to SDS-PAGE and immunoblotting analysis using anti-cyt *c* 7H8.2C-12 (BD-Biosciences, San Jose, CA, USA) or anti-Bax 2D2 monoclonal antibodies (Santa Cruz Biotechnologies, Santa Cruz, CA, USA).

### Steady-state fluorescence spectroscopy

Fluorescence intensity and spectral analyses were done in an 8100 Aminco-Bowman luminescence spectrometer (Spectronic Instruments, Rochester, NY), in thermostatically controlled 4 × 4-mm quartz cuvettes, at 25 °C. Trp spectra were recorded between 305 nm and 405 nm at a scan rate of 1 nm/s, using an excitation wavelength of 295 nm (slits 4 nm). NBD fluorescence spectra in the presence of MOM-like LUVs and proteins of choice were recorded between 500 nm and 620 nm at a scan rate of 1 nm/s, using an excitation wavelength of 465 nm (slits 4 nm). To minimize vesicle light scattering, a 490 nm cut-off filter was placed in the emission light path. In all cases, the signal from background samples (buffer or LUVs in buffer) was substracted from the sample fluorescence. λ_max_ values were determined from the first derivative of the smoothed spectra. F_Q=Dox_ was obtained using MOM-like LUVs containing 20 mol% doxylated lipids substituting equivalent amounts of PC. F_Q=I_- was obtained after addition of 200 mM KI + 0.2 mM Na_2_SO_4_, and sample fluorescence in the absence of quencher (F_0_) was obtained from equivalent samples to which 200 mM KCl + 0.2 mM Na_2_SO_4_ was added. Unless otherwise stated, proteins and LUVs were incubated for 1 h before NBD fluorescence measurements. Release of LUV-encapsulated ANTS/DPX was monitored with λ_ex_ = 350 nm, and λ_em_ = 520 nm (slits, 8 nm). The extent of marker release was quantified on a percentage basis, 15 min after cBID addition, according to the equation: (*F*_*t*_ *−* *F*_0_/*F*_100_ − *F*_0_) × 100, where *F*_*t*_ is the measured fluorescence of protein-treated LUVs at time *t*, *F*_0_ is the initial fluorescence of the LUV suspension before protein addition, and *F*_100_ is the fluorescence value after complete disruption of LUVs by addition of C_12_E_8_ detergent (0.5 mM). BAX, cBID, BCLXL, and BCLXLΔC concentrations were 200 nM, 50 nM, 500 nM, 5000 nM, respectively. Lipid concentration was 200 μM.

### Monolayer surface pressure measurements

Measurements were carried out with a MicroTrough-S system from Kibron (Helsinki, Finland) at 25 °C with constant stirring. The MOM-like lipid mixture, dissolved in chloroform, was gently spread over the surface and kept at a constant surface area. The desired initial surface pressure, π_i_, was attained by changing the amount of lipid applied to the airwater interface. After 10 min to allow for solvent evaporation, the peptide (1 μM) was injected through a hole connected to the subphase. The change in surface pressure, Δπ, was recorded as a function of time until a stable signal was obtained. The linear plot of Δπ as a function of π_i_ can be extrapolated to a π_i_ of 0 to give the critical pressure, π_c,_ which is a measure of the relative “penetration capacity” of a protein into the monolayer.

### 31P NMR Measurements

Samples for 31P NMR were prepared by dispersing 15 μmol of dry MOM-like lipid mixtures in 0.5 ml of KHE buffer alone or containing the peptide of interest (0.15 μmol). Multilamellar vesicle suspensions were freeze-thawed 3 times in liquid N_2_ to disperse the added proteins in the lipid membranes, and the liposomes were spun down in an Eppendorf centrifuge (14000 *g*, 15 min, 4 °C). Pellets were loaded directly into 5-mm Pyrex NMR tubes. High power, proton noise-decoupled 31P NMR spectra were recorded at 25 °C on a Bruker AV-500 spectrometer operating at 202.4 MHz using 5-mm broadband inverse probes with z-gradient equipment. 1024 free induction decays were averaged using a 2-s recycle delay. Spectra were processed and evaluated using TOPSPIN 1.3 (Bruker) and plotted with 80-kHz line broadening.

### Computational tools and simulations

BAX and BCLXL structural representations were generated by Pymol. Main biophysical characteristics of BAX-derived peptides were determined by MPEx^39^ and Heliquest^40^. MC simulations of the interaction of peptide molecules with MOM-like membranes were performed using the MCPep web server^42^. The web server´s output includes the free energy of peptide-membrane association, the energetically favorable peptide-membrane depth of penetration and tilt, and snapshots of example simulations with the centroid conformation of the largest cluster (in PDB format).

## Electronic supplementary material

Supplementary Information
