# Supplementary material for: Membrane insertion of the BAX core, but not latch domain, drives apoptotic pore formation
Source: Sci Rep. 2017 Nov 24;7:16259. doi: 10.1038/s41598-017-16384-4 (PMC5701199; doi:10.1038/s41598-017-16384-4)
Supplement: Supplementary file 1 — Supplementary Information [file 41598_2017_16384_MOESM1_ESM.pdf]

Supplementary Information:

Membrane insertion of the BAX core, but not latch domain, drives apoptotic pore formation

Authors: Hector Flores-Romero[1], Miguel Garcia-Porras[1] & Gorka Basañez[1*]

[1] Biofisika Institute (CSIC, UPV/EHU), Barrio Sarriena s/n, Leioa, 48940, Spain.

*Correspondence and requests for material should be addressed to G. B. (email: gorka.basanez@ehu.eus)

Supplementary Materials and Methods

DSF assays. Fluorescence measurements were made in a Fluorolog spectrofluorimeter (Horiba Jobin Yvon, Bensheim, Germany) equipped with a Peltier LFI-3551, using 1-ml cuvettes with continuous stirring. Measurements were set up such that the samples would initially equilibrate at 25 °C and then increased up to 95 °C in steps of 1 °C, holding the temperature at each step for 60 s. We used 4,4′-Dianilino-1,1′-binaphthyl-5,5′-disulfonate (bis-ANS, Sigma) as the fluorescent dye, with λ_{ex}=390 nm and λ_{em}=490 nm. Bis-ANS stock solutions for the DSF measurements were prepared in KHE on the day of measurement. Final Bis-ANS and protein concentrations were 10 μM and 1 μM, respectively. To determine the melting temperature (T_m), a modified Boltzmann equation is fit to fluorescent data. Before T_m were determined, a baseline approximation was subtracted from the fluorescence using the dye alone with no protein.

Supplementary Figure S1.

Cyt c-releasing capacity and DSF behaviour of monocysteine BAX mutants, as compared to BAX wt. (A) Mitochondria isolated from $BAX^{-/-}$/$BAK^{-/-}$DKO MEFs were treated with BAX wt or indicated BAX monocysteine mutants, with or without cBID, followed by separation of supernatant and pellet fractions, SDS-PAGE analysis, and immunoblotting for cyt c. Left: cropped western-blotting images, Right: uncropped western-blotting images. **(B)** Representative DSF melting curves for BAX and indicated monocysteine BAX variants. Data were normalized by dividing fluorescence signal by the maximum fluorescence intensity achieved during the run. **(C)** Melting point values (T_m) and change in Tm relative to wt BAX (ΔTm) were obtained by fitting fluorescent data to a modified Boltzmann equation. Error bars represent the SDs of the mean obtained from three independent experiments.

A

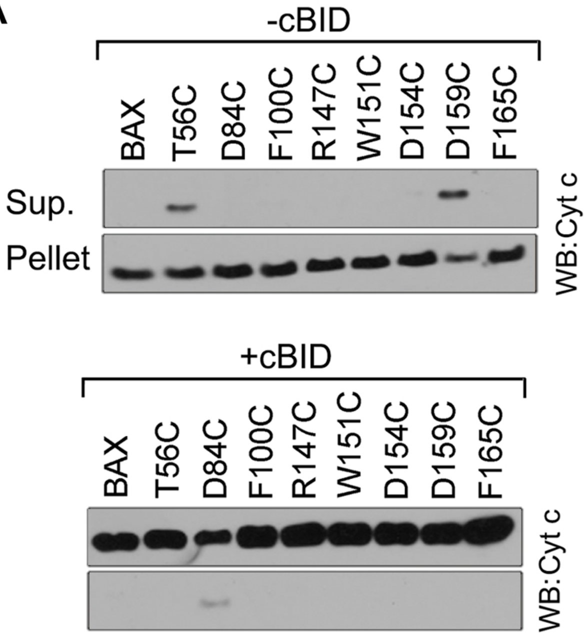

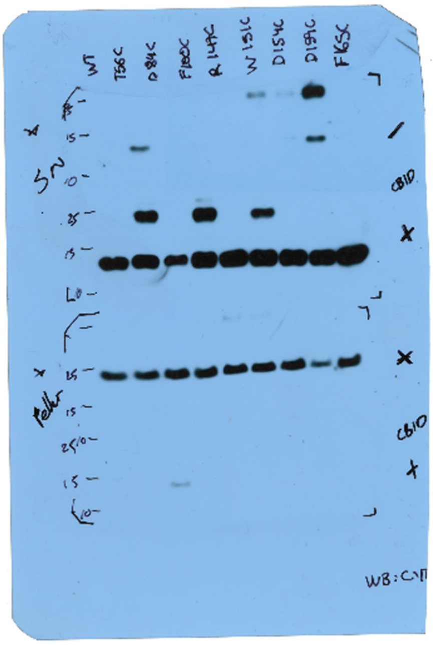

B

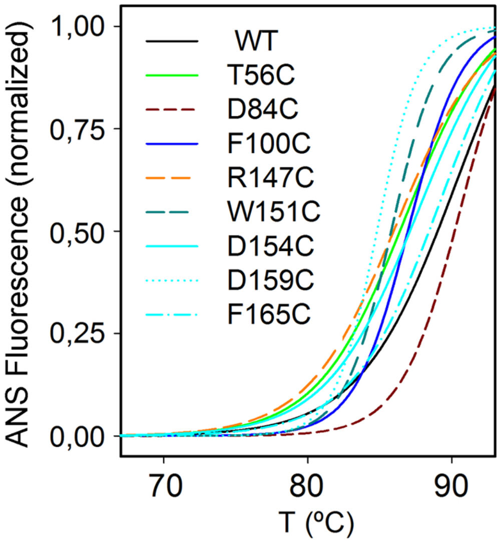

C

Mutant	Tm (˚C)	S.E.	ΔTm (˚C)
WT	90,4055	1,7914	0
T56C	86,7753	0,8953	-3,63
D84C	90,6743	1,2572	+0,27
F100C	86,9957	0,7979	-3,41
R147C	86,1922	0,7137	-4,21
W151C	85,8026	0,6038	-4,60
D154C	87,6591	1,0109	-2,75
D159C	84,8646	0,537	-5,54
F165C	89,2297	1,5559	-1,18

Supplementary Table 1: Thermodynamic characteristics of interaction between different peptides and MOM-like membranes.

Peptide/Quality	ΔG_{total} (kT)	Z_{center} (Å)	Tilt (degrees)
Glycophorin A (TM)	-40.8±0.3	2.8±0.4	21.3±0.7
Endophilin H0	-7.7±0.1	22.4±0.7	68.3±0.6
Melittin	-22.9±0.2	18.5±0.3	79.4±0.5
BAX α5	-26.1±0.3	18.1±0.1	78.9±0.3
BAX α6	-3.7±0.4	36.2±0.7	58.0±0.4
BAX α7-α8	-0.8±0.5	45.3±0.6	53.8±0.4
BAX α9	-17.7±0.1	0.8±0.1	11.5±0.4

All values are reopreted as means ± standard error. ΔG_{total}, the total free energy of peptide-membrane association; Z_{center}, the average distance of the peptide´s center of mass to the membrane midplane; Tilt, the angle between the N´-to-C´vector of the peptide´s helical core and the membrane normal.
